# Whipple's procedure for pancreatic cancer: training and the hospital environment are more important than volume alone

**DOI:** 10.1016/j.sipas.2023.100211

**Published:** 2023-08-15

**Authors:** Shamir O. Cawich, Robyn Cabral, Jacintha Douglas, Dexter A. Thomas, Fawwaz Z. Mohammed, Vijay Naraynsingh, Neil W. Pearce

**Affiliations:** a Department of Clinical Surgical Sciences, University of the West Indies, St Augustine Campus, Trinidad & Tobago, West Indies; b Department of Surgery, Southampton University NHS Trust, Southampton, United Kingdom SO16DP

**Keywords:** Pancreas, Whipples, Resection, Outcomes, Volume

## Abstract

**Background:**

In our center, patients with pancreatic cancer traditionally had Whipple's resections by general surgery teams until January 2013 when a hepatopancreatobiliary (HPB) was introduced. We compared outcomes before and after introduction of HPB teams.

**Methods:**

Data were collected from the records of all patients booked for Whipple's resections over a 12-year period. The data were divided into two groups: Group A consisted of the 6-year period from January 1, 2007 to December 30, 2012 during which all resections were performed by GS teams. Group B comprised patients in the 6-year period from January 1, 2013 to December 30, 2019 during which operations were performed by HPB teams. All statistical analyses were carried out using SPSS ver 16.0 and a P Value <0.05 was considered statistically significant.

**Results:**

The patients selected for Whipple's resections in Group A had statistically better performance status and lower anaesthetic risk. Despite this, patients in Group A had higher conversions to palliative operations (66% vs 5.3%), longer mean operating time (517±25 vs 367±54 min; P<0.0001), higher blood loss (3687±661 vs 1394±656 ml; P<0.0001), greater transfusion requirements (4.3±1.3 vs 1.9±1.4 units; P<0.001), greater likelihood of prolonged ICU stay (100% vs 40%; P=0.19), higher overall morbidity (75% vs 22.2%; P=0.02), higher major morbidity (75% vs 13.9%; P=0.013), more procedure-related complications (75% vs 9.7%; P=0.003) and higher mortality rates (75% vs 5.6%; P<0.0001). The HPB teams were more likely to perform vein resection and reconstruction to achieve clear margins (26.4% vs 0; P=0.57).

**Conclusion:**

This paper adds to the growing body of evidence that volume alone should not be used as a marker of quality for patients requiring Whipple's procedures.

## Highlights

• Whipple's procedure is a safe procedure when trained teams perform the procedure.
• Volume alone should not be used as a marker of quality.
• Hospital and Surgeon training result in better outcomes.
• Continuous adaptive learning by the entire hospital is important.

## Introduction

In the English-Speaking Caribbean, pancreatic cancer occurs at an incidence of 4-4.5 per 100,000 persons per annum [1]. It is accepted that a Whipple's procedure is the best therapeutic option for adenocarcinoma at the head of the pancreas [2].

Although it is a complex operation, the Whipple's procedure is considered safe when carried out by trained hepatopancreatobiliary (HPB) teams in specialized centers [2], [3]. Over the past decade there has been a trend to refer these patients to centralized hospitals for HPB teams to perform Whipple's operations in large volumes [4], [5].

At our institution in Trinidad & Tobago, a small island state in the Eastern Caribbean, HPB services were introduced in 2013. Prior to this, all pancreatic resections were performed by General Surgery (GS) teams. We sought to determine whether there were any differences in short term outcomes when pancreatic head adenocarcinomas were treated by HPB vs GS teams.

## Methods

This Caribbean nation has a population of 1.4 million persons [6]. Under the auspices of the Caribbean Chapter of the Americas Hepatopancreatobiliary Association (AHPBA) an attempt was made at service centralization for hepatobiliary diseases with the establishment of three HPB units in 2011 [6]. The General Hospital in Port of Spain houses one of these HPB units [6]. At this facility, a formal HPB unit was incorporated in January 2013. This HPB unit was comprised of 2 fellowship-trained HPB surgeons, one specialized anaesthetist, a dedicated senior registrar and 2 junior residents. All cases were discussed in multidisciplinary team meetings where therapeutic decisions were made. Prior to incorporation of the HPB team, all liver and pancreatic resections at this institution were performed by GS teams.

We secured institutional review board approval to collect data from all patients who underwent Whipple's procedures at this facility. We sought to collect data for a period of 12 years, from January 1, 2007 to December 30, 2019. This period was deliberately chosen to compare short-term outcomes before and after the January 2013 date when the HPB service was incorporated.

We retrospectively collected data from the registers at the pathology department, operating theatres, ICU department and MDT records to identify all consecutive patients with pancreatic head adenocarcinomas during the study period. The subset of patients who were booked for Whipple's resections were identified and their hospital records were retrieved for detailed review.

We excluded patients who were not candidates for Whipple's resections, those whose paper-based records could not be retrieved, patients who underwent intra-operative conversions to palliative bypass procedures and those who underwent laparoscopic Whipple's operations.

The following data were extracted: patient demographics, performance status using the Eastern Cooperative Oncology Group (ECOG) classification, physical status using the American Society of Anaesthesiologists (ASA) scoring system, estimated operative blood loss, duration of operation (from incision to closure), post-operative complications, 30-day mortality, completion of Whipple's operations, conversions to surgical palliation, duration of hospitalization and transfusion requirements.

Complications were classified using the modified Clavien-Dindo system [7]. Pancreatic leak was categorized according to the International Study Group on Pancreatic Fistula (ISGPF) criteria [8]. Massive operative bleeding was defined as the loss of >1 blood volume within the operative period or 50% of the patient's blood volume in <3 hours [9]. Massive transfusion was defined as receipt of >10 units of packed red cells in 24 hours or >6 units within 6 hours [10].

Complications were also subdivided into medical and procedure-related complications. Medical complications include aspirations, pneumonia, pulmonary failure, deep vein thromboses, pulmonary embolus, myocardial infarction, arrhythmias, congestive heart failure, renal failure and septicemia [11], [12]. Procedure-related complications include massive intra-operative bleeding, pancreatic fistula, delayed gastric emptying, surgical site infection, organ space collection, pseudoaneurysm and post-operative haemorrhage [11], [12].

The records of all patients who were booked for Whipple's resections were retrieved and data were divided into two groups: Group A consisted of the 6-year period from January 1, 2007 to December 30, 2012 during which all resections were performed by GS teams. Group B consisted of patients in the 6-year period from January 1, 2013 to December 30, 2019 during which operations were performed by HPB teams.

All data were compared between the two groups. The t-test for independent means was used to compare continuous numerical values between the groups. The Chi Square and test were used to compare categorical variables between the groups. Statistical analyses were carried out using SPSS ver 16.0 and a P Value <0.05 was considered statistically significant.

## Results

Over the 12-year study period, 85 patients were diagnosed with potentially operable pancreatic head carcinomas. Data from patients with pancreatic adenocarcinoma who were not candidates for Whipple's procedures were excluded.

In Group A, there were 9 patients with potentially resectable pancreatic head adenocarcinomas who were booked for Whipple's procedures. There were 4 males and 5 females at a mean age of 54.7 years (SD ±7.5; Range 44-65; Median 55). In this group, 5 (56%) patients were booked for Whipple's procedures but deemed to have irresectable disease at surgical exploration. In these patients, the operative procedures were converted to surgical palliative bypasses. These patients were excluded from further analyses. Four (44%) patients had completion of the planned Whipple's procedure. In this group, the mean age was 50.8 years (SD ±7.4; range 44-59; Median 50). Tables 1 and 2 document the ECOG scores and ASA scores in this group.

**Table 1:** ASA Scores for Patients Undergoing Whipple's Procedures.

Score	ASA Descriptor	GS	HPB	P
I	Completely healthy	**4 (100%)**	**10 (13.9%)**	**0.0006**
II	Mild Systemic Disease	0	24 (33.3%)	-
III	Severe Systemic Disease, not incapacitating	0	30 (41.7%)	-
IV	Incapacitating disease that is a threat to life	0	8 (11.1%)	-
V	Moribund and not expected to survive > 24 Hours	0	0	-

**Table 2:** Performance Scores for Patients Undergoing Whipple's Procedures.

Grade	ECOG Performance Status	GS	HPB	P
0	Fully active, able to carry out all activities without restriction	**4 (100%)**	**13 (18.1%)**	**0.0015**
1	Restricted in physically strenuous activity, but ambulatory and able to carry out light work	0	20 (27.8%)	-
2	Ambulatory and capable of self-care, but unable to carry out work activities. Up and about >50% of waking hours	0	34 (47.2%)	-
3	Capable of limited self-care and confined to bed or chair for more than 50% of waking hours	0	4 (5.6%)	-
4	Completely disabled and cannot carry on self-care. Confined to bed or chair	0	1 (1.4%)	-
5	Dead	0	0	-

In Group B, 76 patients with pancreatic head carcinomas were booked for Whipple's procedures. There were 34 males and 42 females at a mean age of 60.2 years (SD±9.28; range 46-77; Median 61). In this group, 4 (5.3%) patients were deemed irresectable at the time of operation and underwent palliative bypasses. These patients were excluded from the final sample of 72 patients who had completion of Whipple's procedures. Tables 1 and 2 document the ASA and ECOG scores in this group.

### Operative details

In Group A, the mean operating time for Whipple's procedures was 517.5 minutes (Range 490-550; SD±25; Median 515). The operations in these patients were accompanied by a mean blood loss of 3687.5 ml (Range 2500-5000; SD± 661.44; Median 3500) and mean packed cell transfusion requirements of 4.25 units (Range 3-6; SD ± 1.26; Median 4). There were no vascular resections or reconstructions performed in this group.

In Group B, the mean operating time was 367 minutes (Range 260-485; SD±54.1; Median 350). The operations in these patients were accompanied by a mean blood loss of 1394 ml (Range 600-4000; SD±656.8; Median 1200) and mean transfusion requirements of 1.88 units of packed cells (Range 0-5; SD ±1.43; Median 2). Nineteen (26.4%) patients underwent planned resections and reconstruction of the superior mesenteric/portal vein. Operative details are compared in Table 3.

**Table 3:** Operative Details in Patients Undergoing Whipple's Resections.

Parameter	GS	HPB	P
Number of patients booked for PD	11	76	
Conversions to palliative bypasses	7 (63.6%)	4 (5.26%)	**<0.0001**
Completed Whipple's resections	4 (36.4%)	72 (94.7%)	**<0.0001**
Planned vascular resections / reconstruction	0	19 (26.4%)	0.5667
Mean operating time in mins (mean ±SD)	517.5±25	367±54.1	**<0.0001** t-value 5.51092
Estimated blood loss in mls (Mean ±SD)	3687.5±661.44	1394±656.8	**<0.0001** t-value 6.60718
Transfusion requirements in units (Mean ±SD)	4.25±1.26	1.88±1.43	**0.000894** t-value 3.24056

### Hospitalization details

In this setting, we maintained a policy of mandatory ICU admission after Whipple's resection because institutional limitations generally did not allow the expected level of supportive care outside of the ICU setting.

In Group A, the mean ICU stay was 12 days (Range 6-12; SD±2.65; Median 8). All (100%) patients required a prolonged ICU stay >72 hours for invasive treatment, ventilator and/or inotropic support. One patient in this group survived to discharge after 17 days of hospitalization.

In Group B, the mean ICU stay of 5.24 days (Range 1-40; SD±7.22; Median 3). Twenty-nine (40.3%) patients required a prolonged ICU stay beyond 72 hours for invasive treatment, ventilator and/or inotropic support. Overall, the mean duration of hospitalization after Whipple's procedure was 15.1 days (Range 8-60; SD±9.53; Median 12). The results are compared in Table 4.

**Table 4:** Hospitalization in Patients Undergoing Whipple's Resections.

Parameter	GS	HPB	P
I.C.U admission in days (Mean ±SD)	12± 2.65	5.24±7.22	0.326408 *t*-value 0.45169
Prolonged ICU stay >72 Hours (Mean SD)	4 (100%)	29 (40.3%)	**0.01878**
Total hospital stay in days (Mean ±SD)	17 *	15.1±9.53	-

⁎ Calculation inappropriate with a single value.

### Morbidity / mortality analysis

Three (75%) patients experienced at least one complication in Group A. Major complications occurred in 3 (75%) patients, all resulting in death within 30-days of operation. These deaths were due to shock secondary to massive intra-operative haemorrhage (1), myocardial infarction (1) and respiratory failure secondary a pneumonia (1).

In Group B, 16 (22.2%) patients experienced at least one complication. Minor complications were recorded in 6 (8.3%) patients and major complications in 10 (14.0%) patients. The individual complications are outlined in Table 5. There were 4 (5.5%) deaths in Group B, due to massive bleeding from a pseudoaneurysm that could not be controlled at re-operation (1), septic shock secondary to biliary sepsis (1), anastomotic leak with intra-abdominal sepsis and multiple organ failure (1) and a massive myocardial infarction (1).

**Table 5:** Complications after Whipple's Procedures.

Morbidity	Description	GenSx	HPB	P
**Overall**	**Number of patients with any complication**	**3** **(75%)**	**16 (22.2%)**	**0.018**
**Minor**	**Clavien-Dindo I or II**	**1 (25%)**	**6 (8.3%)**	0.262
	• Pneumonia	1	2	
	• Deep Vein Thrombosis	0	1	
	• Delayed gastric emptying	0	1	
	• Gastrointestinal bleeding	0	2	
**Major**	**Clavien-Dindo III or IV**	**3 (75%)**	**10 (13.9%)**	**0.013245**
	• Anastomotic dehiscence	0	1	
	• Massive upper GI Bleeding	0	1	
	• Myocardial infarction	1	3	
	• Pseudoaneurysm	1	2	
	• Biliary sepsis as a source of septicemia	0	2	
	• POPF / organ space collection	0	1	
	• Massive Operative Bleeding	1	0	
**Mortality**	30-day mortality	**3 (75%)**	**4 (5.6%)**	**<0.00001**

When medical complications were excluded, significantly more patients in Group A experienced procedure-related complications as outlined in Table 6.

**Table 6:** Procedure-Related Complications after Whipple's Procedures.

Description	GenSx (4)	HPB (72)	P
**Procedure-related complications only**	**3 (75%)**	**7 (9.7%)**	**0.002705**
• Post-operative pancreatic fistula / organ space collection	0	1	
• Massive intra-operative bleeding	1	0	
• Massive transfusion requirement	1	2	
• Delayed gastric emptying	0	1	
• Pseudoaneurysm	1	2	
• Anastomotic leak	0	1	

## Discussion

Whipple's procedure is the only existing treatment with a potential to cure pancreatic head malignancies [13], [14]. Fortuitously, modern series have documented improved complication profiles, with modern 30-day mortality rates between 4-6% [13], [14], [15], [16], [17].

Many factors have been credited as contributors to the improved morbidity profile, including a multidisciplinary approach to care [2], [3], advanced cross-sectional imaging [2,18], specialized surgical equipment [6], ICU availability [19], [20], readily available support services [17], [18], [19], [20] and quaternary surgical training [21]. Prior to the introduction of a HPB service at our facility, advanced cross-sectional imaging, ICU and support services were available. After the introduction of the HPB service in January 2013, a multidisciplinary HPB team was established and met weekly to discuss cases. Specialized surgical equipment was also procured. Although the center remained a low volume center [6], considerable effort was invested to develop hospital-specific protocols geared to the delivery of subspeciality care. We also developed a modified centralization concept that was tailored to our low-volume environment, described in a previous publication [22]. There was good stakeholder buy-in at our institution, and patients requiring Whipple's procedures were referred to the HPB team. This study has shown that there were demonstrable benefits as a result of these changes.

### Case volumes

There was a significant increase in case volumes from 0.7 to 12 Whipple's procedures per annum after introduction of the HPB team. This was likely multifactorial due to (1) changes in referral patterns with increased numbers of patients referred to the service and (2) HPB surgical teams having higher thresholds to convert to surgical palliation because they were more comfortable with complex resections.

Although the case volume increased, we still do not consider this a high-volume center. There is no consensus on the definition of a high-volume center, but most researchers quote numbers >18 Whipple's procedures per annum as high volume [3,13,[23], [24], [25], [26], [27], [28], [29]] and this facility performed a mean of 12 resections annually.

### Resection rates

There was a statistically significant increase in completed resections by HPB surgeons (94.7% vs 36.4%). Considering that resection is the only option that brings a chance for cure, patients with pancreatic cancer are being better served by the HPB teams with greater thresholds for conversion to palliative procedures. We believe this was a reflection of HPB surgeons being more experienced and willing to perform more complex maneuvers, such as portal vein resections, to achieve R0 resections.

### Patient complexity

It was interesting that the patients undergoing resections by GS teams were highly selected. Most teams would operate on patients with ASA scores ≤II and ECOG scores ≤I, but in this study, there was a significant disparity, with all patients in group A having ASA scores I (100% vs 14%) and ECOG scores 0 (100% vs 18.1). It appeared that GS teams had a significantly lower threshold to declare patients medically unfit to undergo Whipple's resections.

The HPB teams were willing to attempt resections in sicker patients. In this group, 53% had ASA scores ≥3 and 54% had ECOG performance scores ≥2. This is an important point as patients with pancreatic carcinoma tend to present late and are more likely to be diagnosed when their decompensation has commenced.

### Operative complexity

Schmidt et al. [3] suggested that the performance of vein resection / reconstruction can be used as a surrogate marker of technical complexity as well as surgeon experience. In this series, more vascular reconstructions were performed by HPB teams (26.4% vs 0). This approached, but did not achieve statistical significance, but it is a clinically important distinction. We agree with Schmidt et al. [3] that it is a complex maneuver, but it is important that pancreatic surgeons are willing and able to perform this maneuver when required to achieve a curative resection.

Schmidt et al. [3] also introduced the concept of the “experienced surgeon” which they defined as one who had performed >50 Whipple's procedures in their career. The surgeons leading the HPB teams in the Caribbean were experienced surgeons having accrued sufficient experience in their career to perform >50 Whipple's procedures. It was not possible for us to compare this metric as there were no statistics available for GS teams.

Considering that HPB surgeons performed more complex resections in patients who were sicker, it was an unexpected finding that operative time, estimated blood loss and transfusion requirements were all significantly greater in Group A. This points to a difference in technical facility between the operating teams.

### Post-operative support services

In this study, there were trends toward shorter duration of ICU stay (5.24 vs 12 days) when HPB teams performed resections, and there was a statistically significant reduction in the likelihood of prolonged ICU stay for invasive treatment / ventilator support in the HPB group (40.3% vs 100%). It was difficult to compare overall hospitalization, since only one patient survived to discharge after 17 days in Group A. These data suggest that the patients, having undergone smoother operations, had less requirement for post-operative support.

### Complication profile

The results have shown that there was a significant reduction in the risk of overall complications (22% vs 75%), major morbidity (14% vs 75%) and mortality (5.6% vs 75%) when Whipple's resections were performed by HPB teams. These results are in keeping with existing data that demonstrated an inverse relationship between case-volumes and overall morbidity [2,3,23] plus 30-day mortality [2,3,23,[30], [31], [32]] following Whipple's resections.

Many authorities have suggested that there is a difference between medical complications and procedure specific complications [11,12]. Medical complications include aspirations, pneumonia, pulmonary failure, renal failure and septicemia [11,12]. Procedure-related complications include pancreatic fistula, delayed gastric emptying, deep surgical site infections, pseudoaneurysms and intra-abdominal haemorrhage [11]. In our series procedure-specific complications were significantly lower in Group B (9.7% vs 50%), and this is reflective of technical expertise. While surgical expertise is necessary, it is insufficient to guarantee good post-operative outcomes [3,11,12]. Medical complications were also greater in group A (50% vs 13.2%), reflective the need for improvement in pre-habilitation.

In Group B, there were experienced surgeons and team development to account for the improved outcomes. However, it is important to note that this is a resource-poor center, with a paucity of blood products, ICU space, operating lists and specialized equipment. This brings special meaning to the fact that good outcomes can be achieved in these settings and outcomes cannot be based on volume data alone.

Recently, there has been a move away from simple volume data toward examining surgeon / team experience using several surrogate markers that include: surgeon experience which was defined as one who had performed >50 Whipple's procedures in their career [3]; technical competence which can be defined by the proportion of vein resection / reconstruction as a surrogate marker [3] and a surpassed learning curve, defined by Tseng et al [33] as >60 Whipple's procedures. By all metrics, the HPB teams in this facility have achieved and surpassed the metrics.

Experience is important for such a technically complex operation. The experienced surgeon would know how to resect / reconstruct the portal vein when required to achieve negative margins [3], when not to operate on patients [34], to recognize aberrant anatomy [34], how to get out of trouble when complications occur intra-operatively [13].

We also believe that two additional factors must be taken into account. Firstly, the surgeon must be willing to adapt to a new working environment with numerous limitations, such as unavailability of ICU space, operating lists, paucity of blood supplies, etc. It is important to adapt practice to focus on peri-operative management and inter-disciplinary cooperation that evolved with time. This interaction and continuous learning are not limited to the surgical team alone. Instead, it involves continuous, adaptive learning by the entire institution [1,2,7,19] by the development of prehabilitation, multidisciplinary team interaction, intra-operative anaesthesia care, surgeon training, post-operative care pathways, post-procedure nursing care, ICU care, availability of emergency medical doctors and experienced subspeciality supportive care [2,3,13,23,[35], [36], [37]].

## Limitations

The retrospective study design inherently introduced a limitation in data collection. Also, because historical data were used retrospectively, it was not possible to evaluate parameters such as training, surgeon experience, etc.

## Conclusion

This paper adds to the growing body of evidence that volume alone should not be used as a marker of quality for patients requiring Whipple's procedures. Despite low volumes at our facility, we demonstrated that all metrics improved when specialty teams were introduced, hospital-based protocols were developed and continuous adaptive learning by the entire hospital were observed. These data support a move away from the traditional volume concept.

## Funding

This research did not receive any specific grant from funding agencies in the public, commercial, or not-for-profit sectors.

## Disclosure statement

There was no funding made available to support this work.

## Declaration of Competing Interest

The authors declare that they have no known competing financial interests or personal relationships that could have appeared to influence the work reported in this paper.
